# Super‐Resolution Ultrasound Radiomics Can Predict the Upstaging of Ductal Carcinoma In Situ

**DOI:** 10.1002/cam4.71155

**Published:** 2025-08-12

**Authors:** Liang Yang, Xiaoxian Li, Zhiyuan Wang, Qian Li, Juan Fu, Xuebin Zou, Xia Liang, Xu Liu, Ruirui Zhang, Junjun Chen, Hui Xie, Yini Huang, Jianhua Zhou

**Affiliations:** ^1^ Department of Ultrasound Sun Yat‐Sen University Cancer Center, State Key Laboratory of Oncology in South China, Collaborative Innovation Center for Cancer Medicine Guangzhou P. R. China; ^2^ Department of Medical Ultrasound The Affiliated Cancer Hospital of Xiangya School of Medicine, Central South University/Hunan Cancer Hospital Changsha Hunan China; ^3^ Department of Ultrasound Affiliated Tumor Hospital of Zhengzhou University, Henan Cancer Hospital Zhengzhou Henan China; ^4^ Department of Ultrasound Dongguan People's Hospital Affiliated to Southern Medical University Dongguan Guangdong China; ^5^ Department of Radiation Oncology Affiliated Hospital (Clinical College) of Xiangnan University Chenzhou Hunan People's Republic of China

**Keywords:** breast cancer, ductal carcinoma in situ, machine learning, super‐resolution reconstruction, ultrasound

## Abstract

**Introduction:**

Preoperatively distinguishing pure ductal carcinoma in situ (DCIS) from upstaged DCIS is important for deciding optimal surgical strategies. However, it is hard to preoperatively predict the upstaging of biopsy‐proven DCIS. This study aims to develop an effective radiomics model for predicting the upstaging of DCIS based on super‐resolution (SR) ultrasound images.

**Methods:**

In this multicentre retrospective study, patients with biopsy‐proven DCIS who underwent ultrasound examination were included. A super‐resolution reconstruction algorithm was used to enhance the resolution of original high resolution (HR) ultrasound images and obtain SR images. Pyradiomics was used for feature extraction. The selected HR radiomics features and SR radiomics features were combined with clinical features to construct the HR fusion model and SR fusion model, respectively. The area under the receiver operating characteristic curve (AUC) of the models and radiologists was analyzed and compared by the Delong test.

**Results:**

A total of 681 women (median age, 47 years; interquartile range, 42–54) with 681 biopsy‐proven DCIS lesions were included, with 422 lesions in the training set, 106 lesions in the validation set, and 153 lesions in the external test set. The SR Fusion model achieved an AUC of 0.819 (0.732–0.887) in the validation set and 0.800 (95% CI 0.728–0.860) in the external test set. It outperformed the radiologists (AUC = 0.603–0.627; *p* < 0.001) in the validation set. Additionally, it surpassed the clinical model (AUC = 0.682, 95% CI 0.602–0.755; *p* = 0.02) and the HR Fusion model (AUC = 0.724, 95% CI 0.646–0.793; *p* = 0.03) in the external test set.

**Conclusion:**

The SR Fusion model integrating SR features and clinical features can effectively predict the upstaging of DCIS.

## 1 Introduction

Ductal carcinoma in situ (DCIS) accounts for approximately 20% of all newly diagnosed breast tumors, and the incidence has been increasing annually as a result of breast cancer screening [1, 2]. Due to the tumor heterogeneity, the pathology results of core needle biopsy (CNB) may not accurately represent the entire lesion [3, 4]. A previous meta‐analysis revealed that 25.9% of patients diagnosed with DCIS via CNB were upstaged to invasive breast cancer (IBC) at surgery [5]. Compared to patients with upstaged DCIS who required axillary surgery to determine lymph node staging, patients with pure DCIS have a lower rate of lymph node metastasis, for whom axillary surgery is unnecessary and can be omitted [6, 7]. Therefore, a method for accurately predicting upstaged DCIS before surgery is needed to identify suitable patients for axillary surgery.

Previous studies have identified specific clinical and pathological factors that are significantly associated with the upstaging of DCIS lesions. However, the predictive models constructed using these factors have shown limited reliability and performance [8, 9, 10]. Imaging examinations can provide more comprehensive information about the entire DCIS lesions compared to CNB. Radiomics, which extracts quantitative metrics that capture lesion characteristics such as heterogeneity and shape within medical images, has shown great potential in diagnosing breast cancer [11]. Previous studies have shown that radiomics based on mammography and MRI can predict upstaged DCIS, with the areas under the receiver operating characteristic curve (AUCs) ranging between 0.7 and 0.8 [12, 13, 14]. Compared to mammography and MRI, ultrasound is radiation‐free, widely available at low cost, and plays an important role in preoperatively evaluating breast lesions, especially in patients with dense breast tissue and in the identification of non‐calcified DCIS [15]. Thus, applying radiomics to ultrasound may provide a valuable tool in predicting the upstaging of DCIS. When evaluating such predictive tools, interpretability and data efficiency are paramount clinical considerations. While deep learning achieves strong performance in image classification tasks, its inherent opacity limits clinical interpretability. Conversely, radiomics provides traceable decision support through quantifiable features and demonstrates superior robustness in small‐sample scenarios compared to deep learning approaches [16].

Ultrasound images acquired in retrospective studies often encounter challenges such as low resolution, artifacts, and noise, which may compromise the accuracy of subsequent analysis [17]. Super‐resolution (SR) reconstruction algorithms have been proposed to enhance ultrasound image quality while preserving original details [18]. A previous study showed that radiomics features extracted from SR images could improve the performance in predicting T‐staging of rectal cancer [19]. Therefore, we hypothesized that the performance of the radiomics model to predict upstaged DCIS could be improved using SR ultrasound images.

This study aimed to develop a model based on SR ultrasound images and clinical factors to predict the upstaging of patients with biopsy‐proven DCIS.

## 2 Materials and Methods

### 2.1 Patients and Data Sets

**FIGURE 1:**
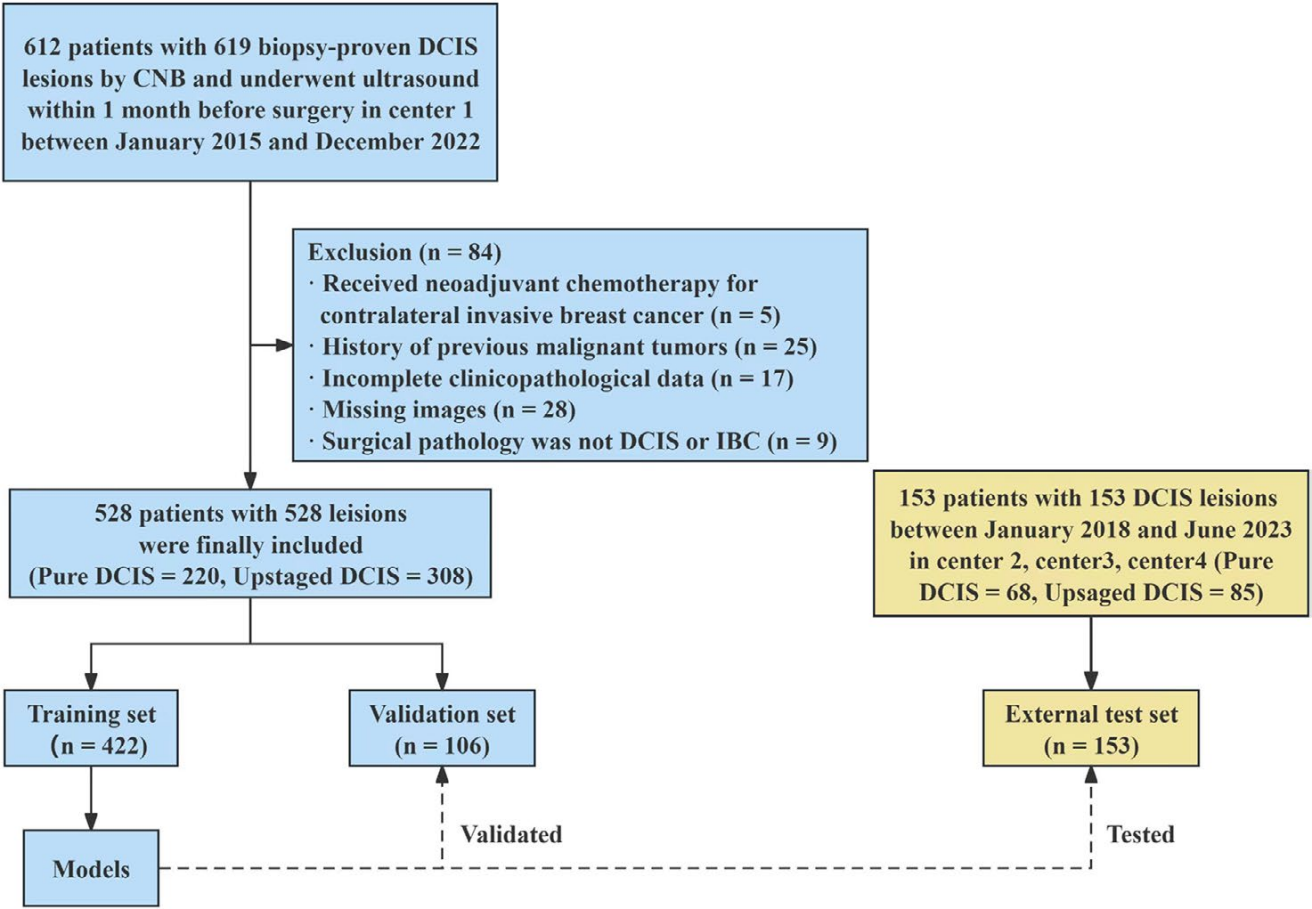
Flowchart of participants' selection. CNB, core needle biopsy; DCIS, ductal carcinoma in situ; IBC, invasive breast cancer.

This retrospective multicentre study was approved by the institutional review board of Sun Yat‐sen University Cancer Center (SYSUCC), with the requirement for informed consent waived. According to the patient inclusion and exclusion criteria, eligible patients acquired from center 1 between January 2015 and December 2021 were included as the training set (80%) and validation set (20%). Additionally, eligible patients acquired from center 2, center 3, and center 4 between January 2018 and June 2023 were included as the external test set. Section 1 in Data S1 provides the inclusion and exclusion criteria. The flowchart of patients' selection is shown in Figure 1. All patients had lesions diagnosed as DCIS by CNB and were categorized into the pure DCIS group (surgical pathology confirmed as DCIS) and the upstaged DCIS group (surgical pathology confirmed as IBC).

Clinical data were obtained from the medical record systems of respective hospitals, including patients' age, lesion size, estrogen receptor (ER) status, progesterone receptor (PR) status, Ki‐67 status, and HER2 status.

### 2.2 Image Collection and Annotation

All collected images were anonymized and acquired using various ultrasound machines (Philips, Siemens, Toshiba, GE Healthcare, Hitachi, Mindray, and Esaote). For each lesion, the image at the plane of the lesion's largest long axis and the image perpendicular to it were utilized. One radiologist (L.Y., with 3 years of experience) manually segmented the regions of interest (ROIs) for all the images, while a second radiologist (X.L., with 4 years of experience) segmented 100 randomly selected images from the training set without prior knowledge of the clinical or histopathological information. The intraclass correlation coefficient (ICC) was used to screen reliable features based on 100 lesions independently segmented by two radiologists (L.Y. and X.L.). The ICC was calculated in SPSS software (version 25.0) using a single‐rater, absolute‐agreement, two‐way random‐effects model. The ROIs were delineated using 3D Slicer software (version 5.6.1).

### 2.3 Super‐Resolution Images Reconstruction

**FIGURE 2:**
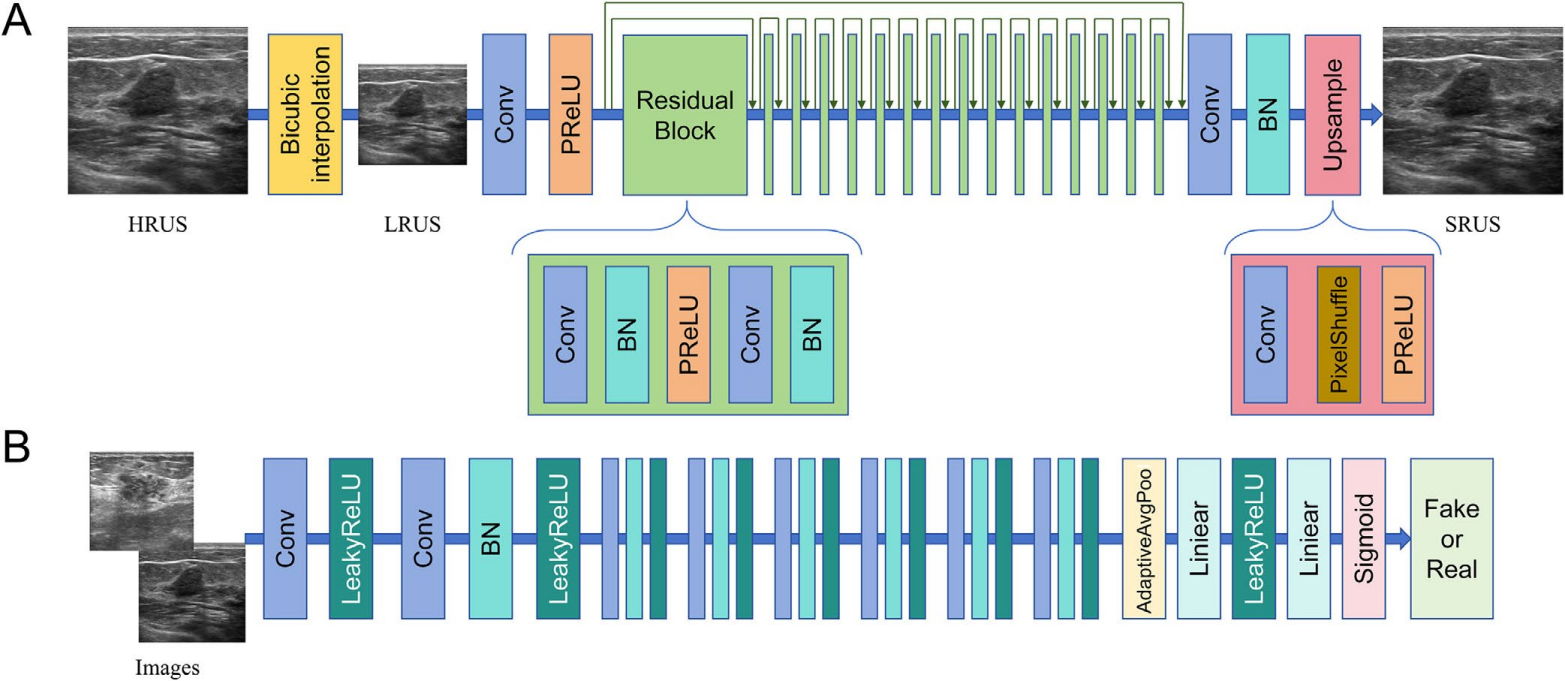
Structural of generator (A) and discriminator (B) in super‐resolution reconstruction algorithm. BN, Batch Normalization; Conv, Convolutional Layer; HRUS, High Resolution Ultrasound images; Leaky ReLU, Leaky Rectified Linear Unit; LRUS, Low Resolution Ultrasound images; PReLU, Parametric Rectified Linear Unit; SRUS, Super Resolution Ultrasound images.

A pre‐trained SR reconstruction model, fine‐tuned on independent breast ultrasound datasets, was employed to process the original high‐resolution ultrasound images (HRUS). The model doubled the size of each image dimension in the training set, generating super resolution ultrasound images (SRUS) with 4 times the original area [20]. The ROI segmentation of the SRUS was automatically generated based on the corresponding ROI of HRUS. The workflow of the SR reconstruction model is shown in Figure 2 and Section 2.1 in Data S1. The image quality after SR reconstruction was evaluated by comparing the HRUS and SRUS using structural similarity (SSIM) and peak signal‐to‐noise ratio (PSNR) [21]. The interpretation of SSIM and PSNR is shown in Section 2.2 in Data S1.

### 2.4 Feature Extraction and Selection

The open‐source Pyradiomics (version 3.1, https://pyradiomics.readthedocs.io), which adheres to the Image Biomarker Standardization Initiative (IBSI), was used for feature extraction within the training set [22, 23]. Feature extraction was performed with the following key parameters: image normalization was applied with a scale of 100; images were resampled to a pixel spacing of [0.2, 0.2] using B‐spline interpolation; the value of bin width was set to 15. While shape features were extracted solely from the original images, other features including first‐order and texture features underwent additional processing using square, square root, wavelet, exponential, local binary pattern, logarithm, and gradient magnitude filters. Ultimately, a total of 1561 features (comprising 306 first‐order features, 14 shape features, and 1241 texture features) were extracted from HR and SR images, respectively (see Table 1; Section 3 and Table S1 in Data S1). Radiomics features with ICC > 0.75 were retained for reliability. Subsequently, redundant features were eliminated by removing one of any pair with Pearson correlation coefficients > 0.9. Finally, the LASSO (Least Absolute Shrinkage and Selection Operator) algorithm was implemented to further identify the most predictive features (see Section 4.1 in Data S1) [24]. The clinical features were selected according to univariable logistic regression analysis (*p* < 0.05).

**TABLE 1:** Radiomics features extracted from ultrasound images.

Filters	First order	Shape	GLCM	GLDM	GLRLM	GLSZM	NGTDM	Total
Original	18	14	22	14	16	16	5	105
Square	18	—	22	14	16	16	5	91
Square root	18	—	22	14	16	16	5	91
Wavelet	144	—	176	112	128	128	40	728
Exponential	18	—	22	14	16	16	5	91
LBP	54	—	66	42	48	48	15	273
Logarithm	18	—	22	14	16	16	5	91
Gradient	18	—	22	14	16	16	5	91
Total	306	14	374	238	272	272	85	1561

*Note:* The numbers in the table represent the number of extracted features. The shape features only extracted from original ultrasound images.

Abbreviations: GLCM, Gray Level Co‐occurrence Matrix; GLDM, Gray Level Dependence Matrix; GLRLM, Gray Level Run Length Matrix; GLSZM, Gray Level Size Zone Matrix; LBP, Local Binary Patterns; NGTDM, Neighboring Gray Tone Difference Matrix.

### 2.5 Model Construction

**FIGURE 3:**
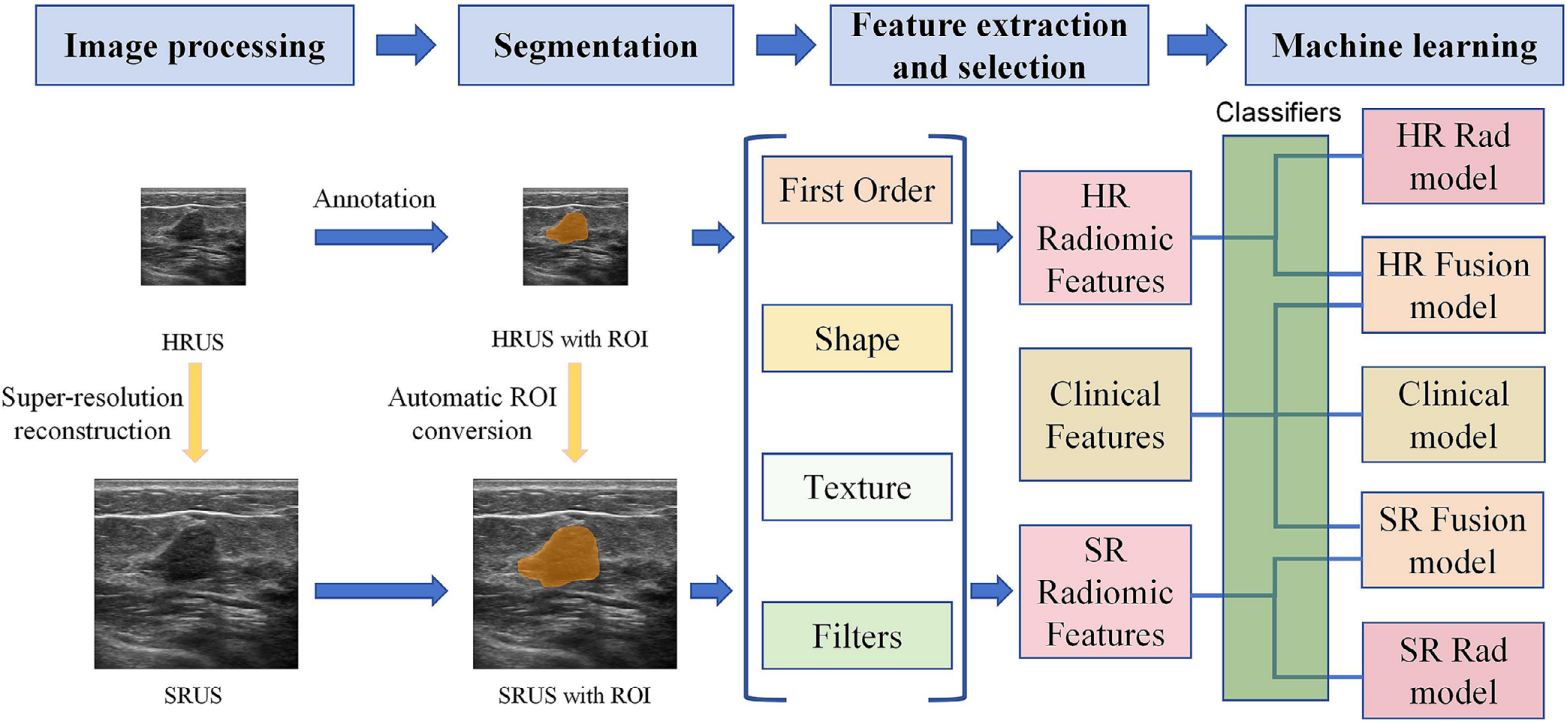
Flowchart of model construction. Original HRUS images are reconstructed using a super‐resolution model to generate SRUS images. The ROI is delineated on the HRUS and then generates the corresponding SRUS image ROI automatically. The radiomics features of HRUS and SRUS images are extracted separately. Subsequently, the selected radiomics features and clinical features were trained in classifiers to obtain the best models. HR Fusion model, model built with clinical and high‐resolution radiomics features; Clinical model, model built with clinical features; SR Rad model, model built with super‐resolution radiomics features; SR Fusion model, model built with clinical and super‐resolution radiomics features. HR Rad model, model built with high‐resolution radiomics features; HRUS, High‐Resolution Ultrasound Images; ROI, region of interest; SRUS, Super‐Resolution Ultrasound Images.

Nine classifiers were trained and validated using five‐fold cross‐validation with grid search on the training set (see Section 4.2 in Data S1). The optimal hyperparameters for each classifier were selected based on the highest mean AUC over the cross‐validation folds (see Table S3). Using these tuned hyperparameters, five distinct models were then formally developed on the full training set. An SR Fusion model, integrating SR radiomics and clinical features, was developed in the training set. Additionally, four other models were developed for comparisons, which were defined as Clinical model (utilizing only clinical features), HR Rad model (utilizing only HR radiomics features), SR Rad model (utilizing only SR radiomics features), and HR Fusion model (integrating HR radiomics and clinical features). Subsequently, for each model type, the best‐performing classifier was selected based on the highest AUC value achieved in the independent validation set. It should be noted that the fusion approach refers specifically to the early concatenation of features prior to model input. The pipeline of this study is shown in Figure 3.

### 2.6 Image Analysis of Radiologists

To evaluate the radiologists' performance in predicting upstaged DCIS, two experienced radiologists (Reader 1: X.Z., with 12 years of experience; Reader 2: J.F., with 9 years of experience) independently reviewed the images in the validation set and classified lesions as either pure DCIS or upstaged DCIS based on typical features such as the tumor's shape, margin, architectural distortion, and calcifications without access to the clinical and pathological data.

### 2.7 Statistical Analysis

Continuous variables were expressed as medians and interquartile ranges and compared using the Kruskal–Wallis test. Categorical variables are expressed as numbers with percentages and compared using the Chi‐squared test. The performance of different models and radiologists was evaluated by AUC, sensitivity, specificity, positive predictive value (PPV), and negative predictive value (NPV). The AUCs were compared by the Delong test [25]. Decision curve analysis was conducted on the validation set and test set to assess the clinical utility of the SR fusion model by quantifying the net benefits across various threshold probabilities. All statistical tests were two‐tailed, and *p* values < 0.05 were considered statistically significant. Statistical analysis was performed using SPSS software (version 25.0, https://www.ibm.com) and R software (version 4.2.3, http://www.Rproject.org).

## 3 Results

### 3.1 Baseline Characteristics

A total of 681 patients with 681 DCIS lesions were included in this study. The training set comprised 422 patients (median age, 47 years; interquartile range, 42–54) with 422 DCIS lesions (247 upstaged DCIS [58%] and 175 pure DCIS [42%]); the validation set comprised 106 patients (median age, 48 years; interquartile range, 43–52) with 106 DCIS lesions (61 upstaged DCIS [57%] and 45 pure DCIS [43%]); and the external test set comprised 153 patients (median age, 49 years; interquartile range, 43–54) with 153 DCIS lesions (85 upstaged DCIS [56%] and 68 pure DCIS [44%]). There were no significant differences in the distribution of clinical and pathological factors among the training set, validation set, and external test set (*p* > 0.05). The details are summarized in Table 2.

**TABLE 2:** Clinical and histopathological characteristics of different datasets.

	Training set (*n* = 422)	Validation set (*n* = 106)	External test set (*n* = 153)	*p*
Upstaged				
Pure DCIS (%)	175 (42)	45 (43)	68 (44)	0.96
Upstaged DCIS (%)	247 (58)	61 (57)	85 (56)	
Median age at diagnosis (years)	47 (42–54)	48 (43–52)	49 (43–54)	0.25
Lesion size (mm)	25 (16–32)	24 (15–32)	25 (16–34)	0.34
ER				
Positive (%)	277 (66)	74 (70)	103 (67)	0.18
Negative (%)	145 (34)	32 (30)	50 (38)	
PR				
Positive (%)	236 (56)	62 (58)	106 (69)	0.05
Negative (%)	186 (44)	44 (42)	47 (31)	
HER2				
Positive (%)	251 (58)	55 (52)	90 (59)	0.53
Negative (%)	171 (41)	51 (48)	63 (41)	
Ki67				
Positive (%)	283 (67)	70 (66)	111 (73)	0.16
Negative (%)	139 (33)	36 (34)	42 (27)	

*Note:* Variables are expressed as numbers of patients with percentages in parentheses, or interquartile ranges in parentheses.

Abbreviations: DCIS, ductal carcinoma in situ; ER, estrogen receptor; HER2, human epidermal growth factor receptor 2; PR, progesterone receptor.

### 3.2 Performance of Super‐Resolution Reconstruction

**FIGURE 4:**
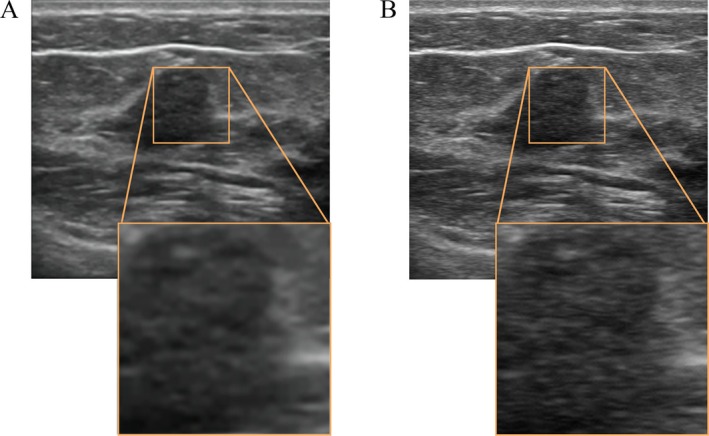
The representative original ultrasound images and their corresponding super‐resolution images from a 43‐year‐old female with pure ductal carcinoma in situ. Original high‐resolution ultrasound images (A) is less blurry compared to super‐resolution images (B) as shown in the orange square box.

The ultrasound images after SR reconstruction achieved an average PSNR of 45.92 dB and SSIM of 0.99, respectively. These values indicate excellent performance in image quality and structural stability after reconstruction. The representative HRUS and the corresponding SRUS are shown in Figure 4.

### 3.3 Feature Selection and Model Building

After feature selection, 5 HR radiomics features, 20 SR radiomics features (Table S2), and 4 clinical features were obtained. The selected clinical features included ER status (OR 0.54 [95% CI 0.33–0.86]), PR status (OR 0.42 [95% CI 0.27–0.65]), Ki67 status (OR 2.09 [95% CI 1.33–3.31]), and lesion size (OR 1.03 [95% CI 1.02–1.06]).

**FIGURE 5:**
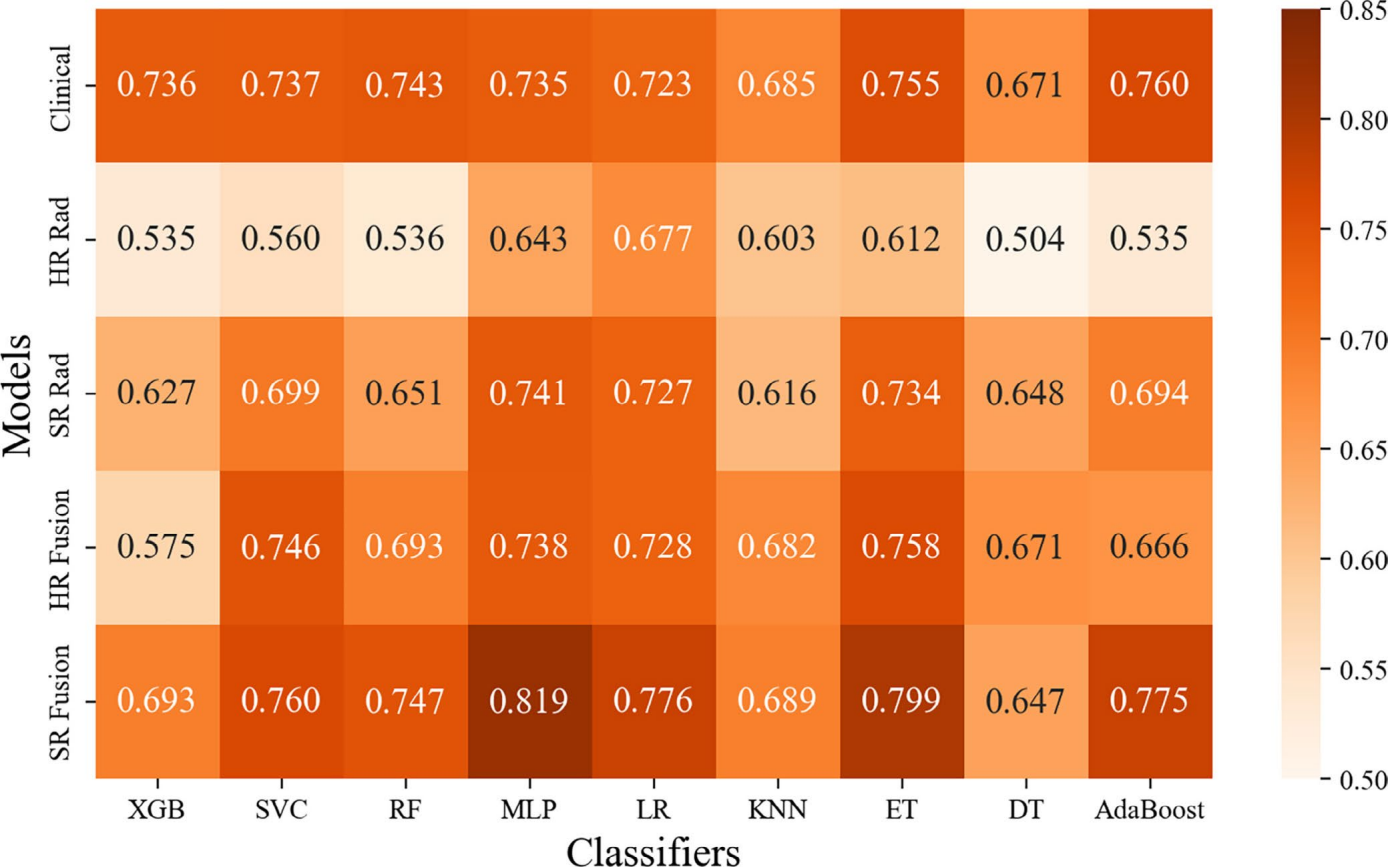
Heat map depicting the AUC value of classifiers (in columns) and models (in rows) in the validation set. AdaBoost, adaptive boosting; DT, decision tree; ET, extremely randomized trees; KNN, K‐nearest neighbors; LR, logistic regression; MLP, multi‐layer perceptron; RF, random forest; SVC, support vector classification; XGB, extreme gradient boosting.

The AUC values of each classifier in the validation sets are presented using heatmaps as shown in Figure 5. Based on the AUC values of each classifier in the validation set, the Adaboost classifier was chosen to construct the Clinical model with an AUC of 0.760, while the LR classifier performed well in constructing the HR Rad model with an AUC of 0.677. The ET classifier was chosen to build the HR Fusion model with an AUC of 0.758. The MLP classifier was chosen to build the SR Rad model (AUC = 0.741) and the SR Fusion model (AUC = 0.819).

### 3.4 The Performance of Different Models

**FIGURE 6:**
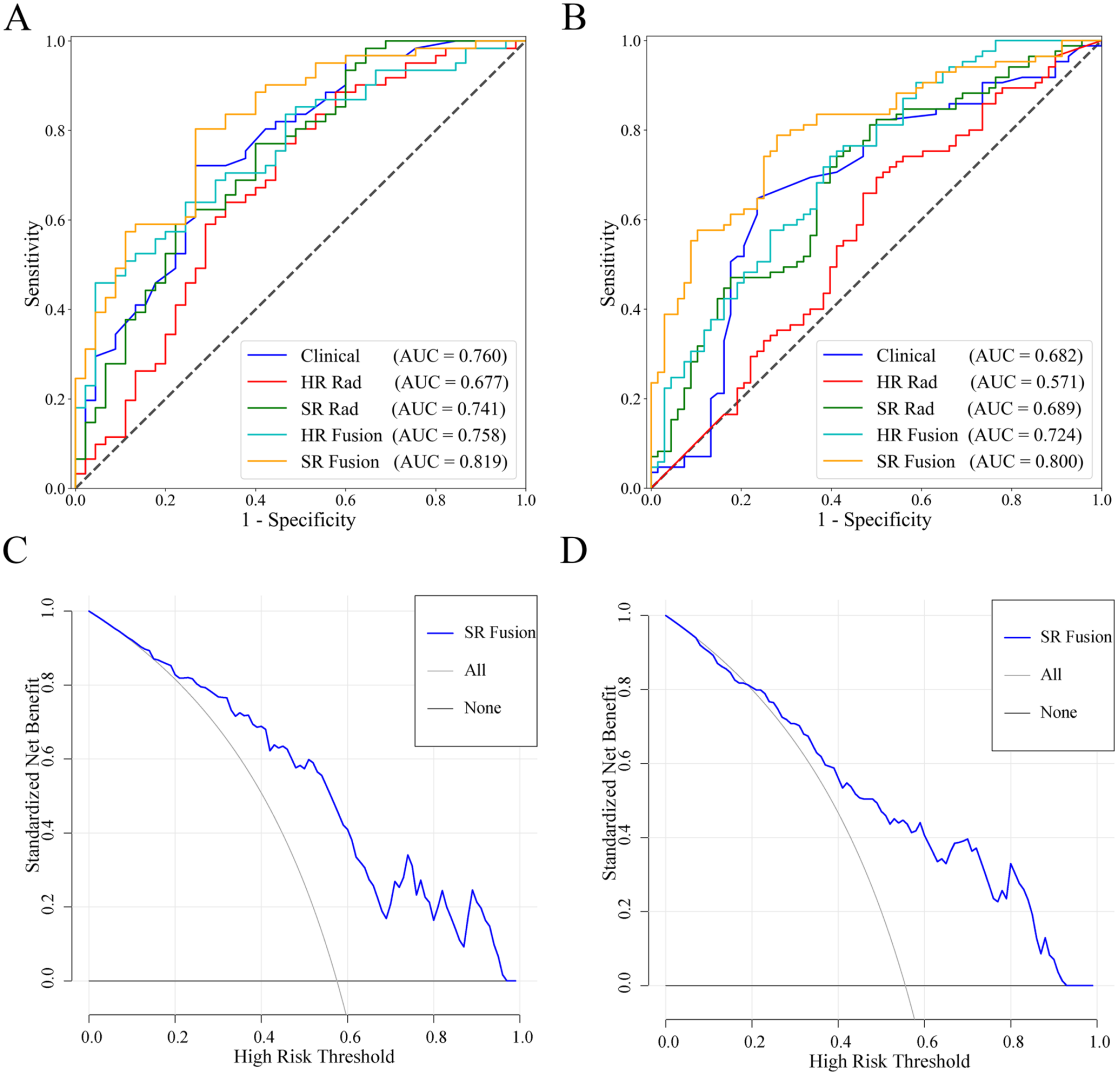
(A, B) Comparison of receiver operating characteristic curves between different models in (A) the validation set and (B) the external test set. (C, D) Decision curve analysis of the SR Fusion model in (C) the validation set and (D) the external test set. AUC, areas under the receiver operating characteristic curves.

The diagnostic performance for predicting the upstaging of DCIS by various models was shown in Table 3 and Figure 6. The AUC of the SR Rad model to distinguish between the pure DCIS and upstaged DCIS in the external test set (AUC, 0.689; 95% CI: 0.610, 0.762) was higher than that of the HR Rad model (AUC, 0.571 [95% CI: 0.488, 0.650; *p* = 0.04]). After integrating clinical features into the radiomics model, the AUC of both the HR Fusion model (AUC, 0.724; 95% CI: 0.646, 0.793) and the SR Fusion model (AUC, 0.800; 95% CI: 0.728, 0.860) in the external test set increased compared with models based on radiomics features alone (*p* = 0.002 for HR Fusion model vs. HR Rad model; *p* = 0.03 for SR Fusion model vs. SR Rad model). The AUC of the SR Fusion model (AUC, 0.800; 95% CI: 0.728, 0.860) was significantly higher than that of the Clinical model (AUC, 0.682 [95% CI: 0.602, 0.755; *p* = 0.02]) and HR Fusion model (AUC, 0.724; 95% CI: 0.646, 0.793; *p* = 0.03) in the external test set.

**TABLE 3:** Performance of models in the training set, validation set, and external test set.

Model	Dataset	AUC	Sensitivity (%)	Specificity (%)	PPV (%)	NPV (%)
Clinical model	T	0.882 (0.847–0.911)	0.907 (0.862, 0.939) [224/247]	0.634 (0.558, 0.705) [111/175]	0.778 (0.724, 0.824) [224/288]	0.828 (0.751, 0.886) [111/134]
	V	0.760 (0.667, 0.837)	0.721 (0.590, 0.825) [44/61]	0.733 (0.578, 0.849) [33/45]	0.785 (0.652, 0.880) [44/56]	0.660 (0.511, 0.784) [33/50]
	E‐T	0.682 (0.602, 0.755)^a^	0.647 (0.535, 0.746) [55/85]	0.765 (0.644, 0.856) [52/68]	0.774 (0.657, 0.861) [55/71]	0.634 (0.519, 0.735) [52/82]
HR Rad model	T	0.762 (0.718, 0.802)	0.809 (0.754, 0.856) [200/247]	0.526 (0.449, 0.601) [92/175]	0.707 (0.649, 0.758) [200/283]	0.662 (0.576, 0.739) [92/139]
	V	0.677 (0.579, 0.764)	0.803 (0.678, 0.890) [49/61]	0.511 (0.360, 0.661) [23/45]	0.690 (0.568, 0.792) [49/71]	0.657 (0.477, 0.803) [23/35]
	E‐T	0.571 (0.488, 0.650)^b^	0.694 (0.583, 0.787) [59/85]	0.500 (0.377, 0.623) [34/68]	0.634 (0.527, 0.730) [59/93]	0.567 (0.433, 0.692) [34/60]
SR Rad model	T	0.790 (0.748, 0.828)	0.923 (0.754, 0.856) [237/247]	0.366 (0.295, 0.442) [64/175]	0.672 (0.619, 0.722) [237/339]	0.771 (0.663, 0.853) [64/83]
	V	0.741 (0.646, 0.821)	0.770 (0.642, 0.865) [47/61]	0.600 (0.444, 0.739) [27/45]	0.723 (0.596, 0.823) [47/65]	0.659 (0.493, 0.794) [27/41]
	E‐T	0.689 (0.610, 0.762)^c^	0.741 (0.633, 0.827) [63/85]	0.588 (0.462, 0.704) [40/68]	0.692 (0.586, 0.783) [63/91]	0.645 (0.513, 0.760) [40/62]
HR Fusion model	T	0.775 (0.953, 0.987)	0.737 (0.676, 0.790) [237/247]	0.577 (0.500, 0.650) [74/175]	0.710 (0.651, 0.765) [182/256]	0.608 (0.529, 0.682) [101/166]
	V	0.758 (0.665, 0.836)	0.459 (0.333, 0.591) [28/61]	0.956 (0.836, 0.992) [43/45]	0.933 (0.765, 0.988) [28/30]	0.566 (0.447, 0.677) [43/76]
	E‐T	0.724 (0.646, 0.793)^d^	0.741 (0.633, 0.827) [63/85]	0.603 (0.477, 0.717) [41/68]	0.700 (0.593, 0.790) [63/90]	0.651 (0.519, 0.764) [41/63]
SR Fusion model	T	0.862 (0.826, 0.894)	0.887 (0.839, 0.922) [219/247]	0.582 (0.505, 0.656) [102/247]	0.750 (0.696, 0.798) [219/292]	0.784 (0.702, 0.850) [102/130]
	V	0.819 (0.732, 0.887)	0.803 (0.678, 0.890) [49/61]	0.733 (0.578, 0.849) [33/45]	0.803 (0.678, 0.890) [49/61]	0.733 (0.578, 0.849) [33/45]
	E‐T	0.800 (0.728, 0.860)	0.788 (0.683, 0.866) [67/85]	0.721 (0.597, 0.819) [49/68]	0.779 (0.674, 0.858) [67/86]	0.731 (0.607, 0.829) [49/67]

*Note:* Data in parentheses are 95% confidence intervals and numbers in brackets are numbers of patients.

Abbreviations: AUC, the area under the curve; CI, confidence interval; E‐T, external test set; NPV, Negative predictive value; PPV, positive predictive value; T, training set; V, validation set.

^a^ *p* = 0.02; Delong et al. in comparison with the SR Fusion model in the external test set.

^b^ *p* = 0.04; Delong et al. in comparison with SR Rad model in external test set.

^c^ *p* = 0.002; Delong et al. in comparison with SR Fusion model in external test set.

^d^ *p* = 0.03; Delong et al. in comparison with SR Fusion model in external test set.

### 3.5 Comparison Between Experienced Radiologists and SR Fusion Model

The performance in predicting the upstaging of DCIS by radiologists was shown in Table 4. The AUCs of the two experienced radiologists were low (0.603 [95% CI: 0.552, 0.654] for reader 1 and 0.627 [95% CI: 0.576, 0.679] for reader 2). There was no difference in AUCs between the two experienced radiologists (*p* = 0.36). The AUC of the SR fusion model was significantly higher than that of the experienced radiologists (*p* < 0.001 for SR fusion model vs. both readers).

**TABLE 4:** Performance of radiologists.

	AUC	Sensitivity (%)	Specificity (%)	PPV (%)	NPV (%)
Reader 1	0.603 (0.552, 0.654)	0.754 (0.624, 0.851) [46/61]	0.511 (0.332, 0.667) [23/45]	0.676 (0.516, 0.781) [46/68]	0.605 (0.423, 0.786) [23/38]
Reader 2	0.627 (0.576, 0.679)	0.508 (0.378, 0.637) [31/61]	0.689 (0.531, 0.813) [31/45]	0.689 (0.532, 0.814) [31/45]	0.508 (0.378, 0.637) [31/61]

*Note:* Data in parentheses are 95% confidence intervals and numbers in brackets are numbers of patients.

Abbreviations: AUC, the area under the curve; CI, confidence interval; NPV, Negative predictive value; PPV, positive predictive value.

### 3.6 Clinical Use

The DCA for the SR fusion model was presented in Figure 6. The DCA showed that if the threshold probability of a patient or doctor was in the range of 0.07–0.96 in the validation set and 0.04–0.93 in the external test set, using the SR fusion model to predict the upstaging of DCIS added more benefit than either the treat‐all‐patients scheme or the treat‐none scheme.

## 4 Discussion

This study established an SR Fusion model that integrates SR radiomics features extracted from reconstructed SR ultrasound images and clinical features to predict the upstaging of DCIS. The SR Fusion model demonstrated good performance with an AUC of 0.800 (95% CI 0.728–0.860); outperforming the HR Fusion model and clinical model in the external test set. The SR Fusion model had superior performance to two experienced radiologists.

Predicting the upstaging of DCIS to IBC before surgery is crucial in determining whether axillary surgery should be performed. However, it is challenging to identify upstaged DCIS via CNB due to tumor heterogeneity. Imaging can provide structural information about the entire lesion, such as the tumor's shape, margin, and architectural distortion, which is valuable in diagnosing breast cancer. Nevertheless, it remains difficult for radiologists to distinguish pure DCIS from IBC based on the limited visual features of images. In this study, the performance of radiologists was relatively low, with AUCs of 0.603–0.627, which is consistent with previous findings [26]. Previous studies have developed predictive models based on clinical factors to distinguish pure DCIS from upstaged DCIS, but these models have shown only limited performance with AUCs around 0.700 [10]. Similarly, the clinical model in this study also demonstrated low performance with an AUC of 0.682. These results highlight the current challenges in preoperatively predicting the upstage of DCIS.

Radiomics enables the extraction of quantitative features from medical images, providing a more detailed analysis than traditional qualitative assessments. Previous studies have shown that radiomics based on MRI and mammography images is feasible in predicting upstaged DCIS, with AUCs of 0.710–0.760 [14, 27]. For women with smaller and denser breast tissue, ultrasound plays an important role in detecting breast cancer [28, 29]. Ultrasound has demonstrated a superior detection rate for non‐calcified DCIS (95%) compared to digital breast tomosynthesis (84%) and digital mammography (68%) [14]. Therefore, constructing a radiomics model based on ultrasound is essential for predicting upstaged DCIS, especially for women with dense breast tissue. In this study, the HR fusion model had a moderate performance with an AUC of 0.724, indicating the potential value of radiomics based on ultrasound and its combination with clinical factors. However, the performance still needs to be improved before it can effectively guide treatment decisions.

Ultrasound images in retrospective studies often suffer from low image quality, which can significantly impact the performance of radiomics models. A previous study showed that SR reconstruction could enhance the performance of the radiomics model in predicting the T‐stage of rectal cancer by improving the quality of CT images [19]. Therefore, this study applied SR reconstruction to ultrasound images and investigated the role of SR ultrasound images in the development of radiomics models. The results demonstrated that the SR reconstruction model could improve the quality of ultrasound images while mitigating the problems of excessive smoothing and loss of details in the reconstructed images. The models constructed using SR ultrasound images were superior to those using HR images (SR Fusion model vs. HR Fusion model, *p* = 0.03; SR Rad model vs. HR Rad model, *p* = 0.04). This suggests that the SR images may better distinguish between DCIS and IBC, providing more relevant information on tumor heterogeneity.

The radiomics features identified in this study demonstrated overlap with previous research, particularly regarding first‐order and texture features [14, 30, 31]. Specific features such as ZoneEntropy, GrayLevelNonUniformity, Correlation, and Skewness were consistently reported across multiple studies, collectively indicating their robustness in characterizing lesion heterogeneity. However, significant variations were observed in feature combinations (e.g., shape features and NGTDM texture features) and the application of filters (e.g., wavelet, square, exponential), which complicate direct cross‐study comparisons. These discrepancies may be due to inherent data heterogeneity (e.g., differences in imaging equipment, acquisition protocols, and segmentation methods) as well as varying study objectives.

This study has several limitations. Firstly, the manual delineation of the ROI by doctors can be a time‐consuming and labor‐intensive process. Secondly, the models exhibited diminished performance in the external test set, which could potentially be attributed to disparities in image quality or data distribution between the internal dataset and the external test set, as well as inadequate generalization ability due to overfitting. Prospective and multiple centers studies are needed to further improve the performance and generalization ability of the model in the future.

## 5 Conclusions

In conclusion, the SR Fusion model integrating SR radiomics features and clinical features can effectively predict the upstaging of biopsied proven DCIS, which may have the potential to facilitate optimal surgical strategies for patients with DCIS based on the core needle biopsy.

## Author Contributions

**Liang Yang:** data curation (equal), formal analysis (lead), investigation (equal), methodology (equal), software (equal), visualization (equal), writing – original draft (lead), writing – review and editing (equal). **Xiaoxian Li:** formal analysis (equal), investigation (equal), methodology (equal), writing – review and editing (equal). **Zhiyuan Wang:** data curation (equal), investigation (equal), validation (equal), writing – review and editing (supporting). **Qian Li:** data curation (equal), investigation (equal), validation (equal), writing – review and editing (supporting). **Juan Fu:** data curation (equal), investigation (equal), validation (equal). **Xuebin Zou:** data curation (equal), investigation (equal), validation (equal). **Xia Liang:** investigation (equal), validation (equal). **Xu Liu:** investigation (equal), validation (equal). **Ruirui Zhang:** investigation (equal), validation (equal). **Junjun Chen:** investigation (equal). **Hui Xie:** methodology (equal), software (equal). **Yini Huang:** conceptualization (equal), data curation (equal), methodology (equal), project administration (equal), supervision (equal), visualization (equal), writing – review and editing (equal). **Jianhua Zhou:** conceptualization (equal), funding acquisition (equal), methodology (equal), project administration (equal), resources (equal), supervision (equal), validation (equal), writing – review and editing (lead).

## Conflicts of Interest

The authors declare no conflicts of interest.

## Supporting information


**Data S1:** cam471155‐sup‐0001‐DataS1.docx.
**Table S1:** Detailed description of radiomics features.
**Table S2:** Selected features by LASSO regression.
**Table S3:** Optimal hyperparameter configurations.
**Figure S1:** Flowchart of features selection. HR, high resolution; SR, super resolution, ICC, intraclass correlation coefficient; LASSO, least absolute shrinkage and selection operator.
**Figure S2:** Feature selection using LASSO algorithm. A and B show the cross‐validation plot for the penalty term and the LASSO path plot of the HR features. C and D show the cross‐validation plot for the penalty term and the LASSO path plot of the SR features. LASSO, least absolute shrinkage and selection operator; HR, high resolution; SR, super resolution.

## Data Availability

The data that support the findings of this study are available from the corresponding authors upon reasonable request.
