# Supplementary material for: Super‐Resolution Ultrasound Radiomics Can Predict the Upstaging of Ductal Carcinoma In Situ
Source: Cancer Med. 2025 Aug 12;14(15):e71155. doi: 10.1002/cam4.71155 (PMC12340541; doi:10.1002/cam4.71155)
Supplement: Supplementary file 1 — Data S1: cam471155‐sup‐0001‐DataS1.docx. Table S1: Detailed description of radiomics features. Table S2: Selected features by LASSO regression. Table S3: Optimal hyperparameter configurations. Figure S1: Flowchart of features selection. HR, high resolution; SR, super resolution, ICC, intraclass correlation coefficient; LASSO, least absolute shrinkage and selection operator. Figure S2: Feature selection using LASSO algorithm. A and B show the cross‐validation plot for the penalty term and the LASSO path plot of the HR features. C and D show the cross‐validation plot for the penalty term and the LASSO path plot of the SR features. LASSO, least absolute shrinkage and selection operator; HR, high resolution; SR, super resolution. [file CAM4-14-e71155-s001.docx]

Data S1

[Supplemental Materials 1 2](#_Toc21957)

[Supplemental Materials 2 3](#_Toc26950)

[2.1 The workflow of super-resolution reconstruction model 3](#_Toc28150)

[2.2 Evaluation of super-resolution ultrasound images 4](#_Toc25214)

[Supplemental Materials 3 6](#_Toc31882)

[Table S1 Detailed description of radiomics features 6](#_Toc2346)

[Supplemental Materials 4 9](#_Toc19957)

[4.1 Feature selection for models 9](#_Toc3781)

[Figure S1 Flowchart of features selection. 10](#_Toc28974)

[Figure S2 Feature selection using LASSO algorithm. 11](#_Toc9238)

[Table S2. Selected features by LASSO regression. 12](#_Toc25323)

[4.2 Classifier selection 12](#_Toc11761)

[Table S3. Optimal Hyperparameter Configurations. 13](#_Toc19388)

[References 14](#_Toc30071)

# Supplemental Materials 1

The inclusion criteria of study patients were as follows:

(1) women diagnosed with DCIS by ultrasound-guided CNB;

(2) breast ultrasound was conducted within 2 weeks before the surgery.

The exclusion criteria were as follows:

(1) Patients received neoadjuvant chemotherapy for contralateral invasive breast cancer;

(2) with a history of previous malignant tumors;

(3) with incomplete clinicopathological data;

(4) with missing images;

(5) with surgical pathology indicating diseases other than DCIS or IBC.

# Supplemental Materials 2

## 2.1 The workflow of super-resolution reconstruction model

The super-resolution reconstruction model employed in this study primarily utilized the SRGAN (Super-Resolution Generative Adversarial Network) framework, which comprised two main components: the generator and discriminator (1). Within the generator network, convolutional blocks were initially employed for extracting shallow features, followed by multiple residual blocks for deep feature extraction. Skip connection was used to enhance information flow across layers and prevent gradient vanishing caused by deepening of network depth. Ultimately, deconvolution layers were utilized for upsampling, transforming the feature maps into high-resolution images. Notably, the upscaling factor for this model was set to 2. The discriminator, on the other hand, consisted of a series of convolutional layers and batch normalization layers, with the Leaky ReLU (Rectified Linear Unit) function implemented to mitigate the problem of dead neurons. Finally, the binary classification output was obtained through fully connected layers and a sigmoid function.

To optimize performance for breast ultrasound characteristics, the pre-trained model based on DIV2K dataset was fine-tuned using an external, independent breast ultrasound dataset (non-overlapping with the main study cohort). Low-Resolution (LR) images were obtained through downsampling using bicubic interpolation. The loss function of the generator consisted of two components: content loss and adversarial loss. Content loss was obtained by calculating the Mean Square Error (MSE) between the feature maps extracted from the High-Resolution (HR) images and the LR images, utilizing a pretrained VGG19 network. Adversarial Loss, on the other hand, employed binary cross entropy (BCE) to measure the difference in discrimination outcomes between the image generated by the generator and the original image in the discriminator. The loss function of the discriminator was also calculated using BCE, evaluating the difference in discrimination probabilities between the original images and the generated images.

The loss function of generator and discriminator are defined as follows:

$$L_{D}=-\lambda(\log D(I^{HR})+\log(1-D(G(I^{LR}))))$$

*W* and *H* represent the respective dimensions of the feature maps within the VGG network. *Φ_i_* represents the feature map output obtained by the i-th convolutional layer of the VGG network. The value of *i* was set to 13. The value of *w* was set to 5 * 10^-4^. The value of *λ* was set to 0.5.

During the training phase, the Adam optimizer was employed, utilizing an initial learning rate of 0.0001. The GPU used in the experiment was NVIDIA GeForce RTX 2080TI, and the environment was Python 3.10 and PyTorch 1.7. Subsequently, the trained generator was utilized for conducting super-resolution reconstruction of ultrasound images.

## 2.2 Evaluation of super-resolution ultrasound images

In this study, the structural similarity (SSIM) and peak signal-to-noise ratio (PSNR) were employed as primary metrics to evaluate the quality of the super-resolution ultrasound images (2,3).

SSIM evaluates the structural similarity, luminance, and contrast between the original and reconstructed images. It ranges from 0 to 1, and a value close to 1 indicates a higher similarity between the two images. It is defined as:

$$SSIM=\frac{2\mu_{x}\mu_{y}+C_{1}}{\mu_{x}^{2}+\mu_{y}^{2}+C_{1}}\times\frac{2\sigma_{\text{xy}}+C_{2}}{\sigma_{\text{x}}^{2}+\sigma_{y}^{2}+C_{2}}$$

Where *μ*_x_ and *μ*_y_ represent the mean intensities, *σ*_x_ and *σ*_y_ represent the variances, *σ*_xy_ stands for the covariance, C1 and C2 are constants.

PSNR evaluates the quality of reconstructed images by measuring the ratio between the maximum possible value of the signal and the power of noise that affects the quality of its representation. A PSNR value higher than 30 dB indicates good image quality. It is defined as:

where MAX_I_ is the maximum possible pixel value of the image, and MSE is the mean squared error between the original and reconstructed images, defined as:

$$MSE=\frac{1}{mn}\sum_{i=0}^{m-1} \sum_{j=0}^{n-1} [I(i,j)-K(i,j)]^{2}$$

# Supplemental Materials 3

The radiomics features—including first-order features, shape features, and texture features (derived from Gray Level Co-occurrence Matrix, GLCM; Gray Level Dependence Matrix, GLDM; Gray Level Run Length Matrix, GLRLM; Gray Level Size Zone Matrix, GLSZM; and Neighboring Gray Tone Difference Matrix, NGTDM ), were extracted from the HR and SR images, respectively. The details of these features are shown in the Table S1.

### Table S1 Detailed description of radiomics features

| Feature Type | Feature Name | Feature Number |
| --- | --- | --- |
| First Order | 10Percentile | 18 |
|  | 90Percentile |  |
|  | Variance |  |
|  | Uniformity |  |
|  | Energy |  |
|  | TotalEnergy |  |
|  | Skewness |  |
|  | RootMeanSquared |  |
|  | RobustMeanAbsoluteDeviation |  |
|  | Range |  |
|  | Minimum |  |
|  | Median |  |
|  | Maximum |  |
|  | MeanAbsoluteDeviation |  |
|  | Kurtosis |  |
|  | InterquartileRange |  |
|  | Entropy |  |
|  | Mean |  |
| Shape | Elongation | 14 |
|  | Flatness |  |
|  | LeastAxisLength |  |
|  | MajorAxisLength |  |
|  | Maximum2DDiameterColumn |  |
|  | Maximum2DDiameterRow |  |
|  | Maximum2DDiameterSlice |  |
|  | Maximum3DDiameter |  |
|  | MeshVolume |  |
|  | MinorAxisLength |  |
|  | Sphericity |  |
|  | SurfaceArea |  |
|  | SurfaceVolumeRatio |  |
|  | VoxelVolume |  |
| Gray-Level Co-occurrence Matrix(GLCM) | Autocorrelation | 22 |
|  | ClusterProminence |  |
|  | ClusterShade |  |
|  | ClusterTendency |  |
|  | Contrast |  |
|  | Correlation |  |
|  | DifferenceAverage |  |
|  | DifferenceEntropy |  |
|  | DifferenceVariance |  |
|  | Id |  |
|  | Idm |  |
|  | Idmn |  |
|  | Idn |  |
|  | Imc1 |  |
|  | Imc2 |  |
|  | InverseVariance |  |
|  | JointAverage |  |
|  | JointEnergy |  |
|  | JointEntropy |  |
|  | MaximumProbability |  |
|  | SumEntropy |  |
|  | SumSquares |  |
| Gray-Level Dependence Matrix(GLDM) | DependenceEntropy | 14 |
|  | DependenceNonUniformity |  |
|  | DependenceNonUniformityNormalized |  |
|  | DependenceVariance |  |
|  | GrayLevelNonUniformity |  |
|  | GrayLevelVariance |  |
|  | HighGrayLevelEmphasis |  |
|  | LargeDependenceEmphasis |  |
|  | LargeDependenceHighGrayLevelEmphasis |  |
|  | LargeDependenceLowGrayLevelEmphasis |  |
|  | LowGrayLevelEmphasis |  |
|  | SmallDependenceEmphasis |  |
|  | SmallDependenceHighGrayLevelEmphasis |  |
|  | SmallDependenceLowGrayLevelEmphasis |  |
| Gray-Level Run-Length Matrix(GLRLM) | GrayLevelNonUniformity | 16 |
|  | GrayLevelNonUniformityNormalized |  |
|  | GrayLevelVariance |  |
|  | HighGrayLevelRunEmphasis |  |
|  | LongRunEmphasis |  |
|  | LongRunHighGrayLevelEmphasis |  |
|  | LongRunLowGrayLevelEmphasis |  |
|  | LowGrayLevelRunEmphasis |  |
|  | RunEntropy |  |
|  | RunLengthNonUniformity |  |
|  | RunLengthNonUniformityNormalized |  |
|  | RunPercentage |  |
|  | RunVariance |  |
|  | ShortRunEmphasis |  |
|  | ShortRunHighGrayLevelEmphasis |  |
|  | ShortRunLowGrayLevelEmphasis |  |
| Gray-Level Size-Zone Matrix(GLSZM) | GrayLevelNonUniformity | 16 |
|  | GrayLevelNonUniformityNormalized |  |
|  | GrayLevelVariance |  |
|  | HighGrayLevelZoneEmphasis |  |
|  | LargeAreaEmphasis |  |
|  | LargeAreaHighGrayLevelEmphasis |  |
|  | LargeAreaLowGrayLevelEmphasis |  |
|  | LowGrayLevelZoneEmphasis |  |
|  | SizeZoneNonUniformity |  |
|  | SizeZoneNonUniformityNormalized |  |
|  | SmallAreaEmphasis |  |
|  | SmallAreaHighGrayLevelEmphasis |  |
|  | SmallAreaLowGrayLevelEmphasis |  |
|  | ZoneEntropy |  |
|  | ZonePercentage |  |
|  | ZoneVariance |  |
| Neighbouring Gray-Tone Difference Matrix(NGTDM) | Busyness | 5 |
|  | Coarseness |  |
|  | Complexity |  |
|  | Contrast |  |
|  | Strength |  |

# Supplemental Materials 4

## 4.1 Feature selection for models

The flowchart of feature selection is shown in Figure S1. The initial set of features consisted of 1561 radiomics features and 6 clinical features (including age, lesion size, ER status, PR status, Her-2 status, KI67 status). The radiomics features underwent three screening steps: first, features with an intraclass correlation coefficient (ICC) greater than 0.75 were retained, and then features with a Pearson correlation coefficient greater than 0.9 were excluded. Subsequently, the least absolute shrinkage and selection operator (LASSO) algorithm was employed for further feature selection (Figure S2). 5 HR radiomics features, 20 SR radiomics features was chosen (Table S2). For the clinical features, they were screened using the univariable logistic regression algorithm with a significance threshold of P < 0.05. Finally, a total of 4 clinical features were ultimately chosen for further analysis as follows: ER status, PR status, Ki67 status, and lesion size.


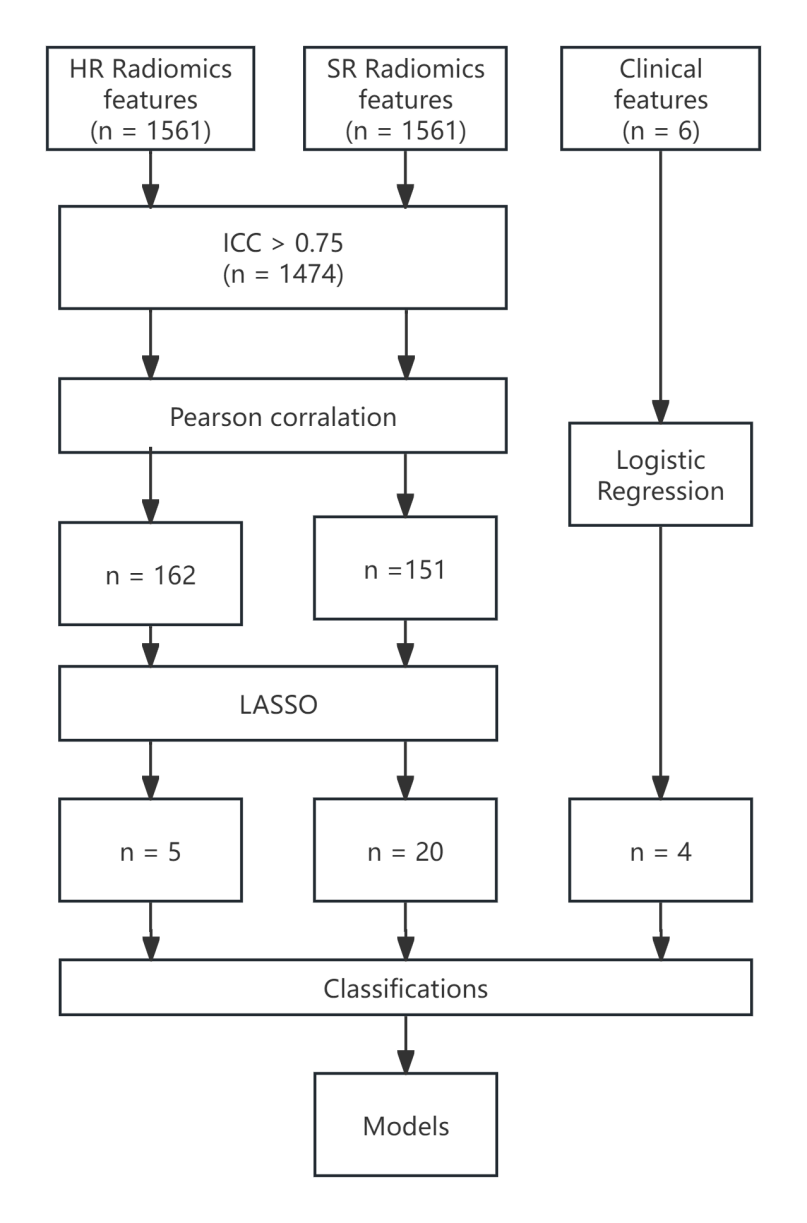


Figure S1 Flowchart of features selection. HR, high resolution; SR, super resolution, ICC, intraclass correlation coefficient; LASSO, least absolute shrinkage and selection operator.


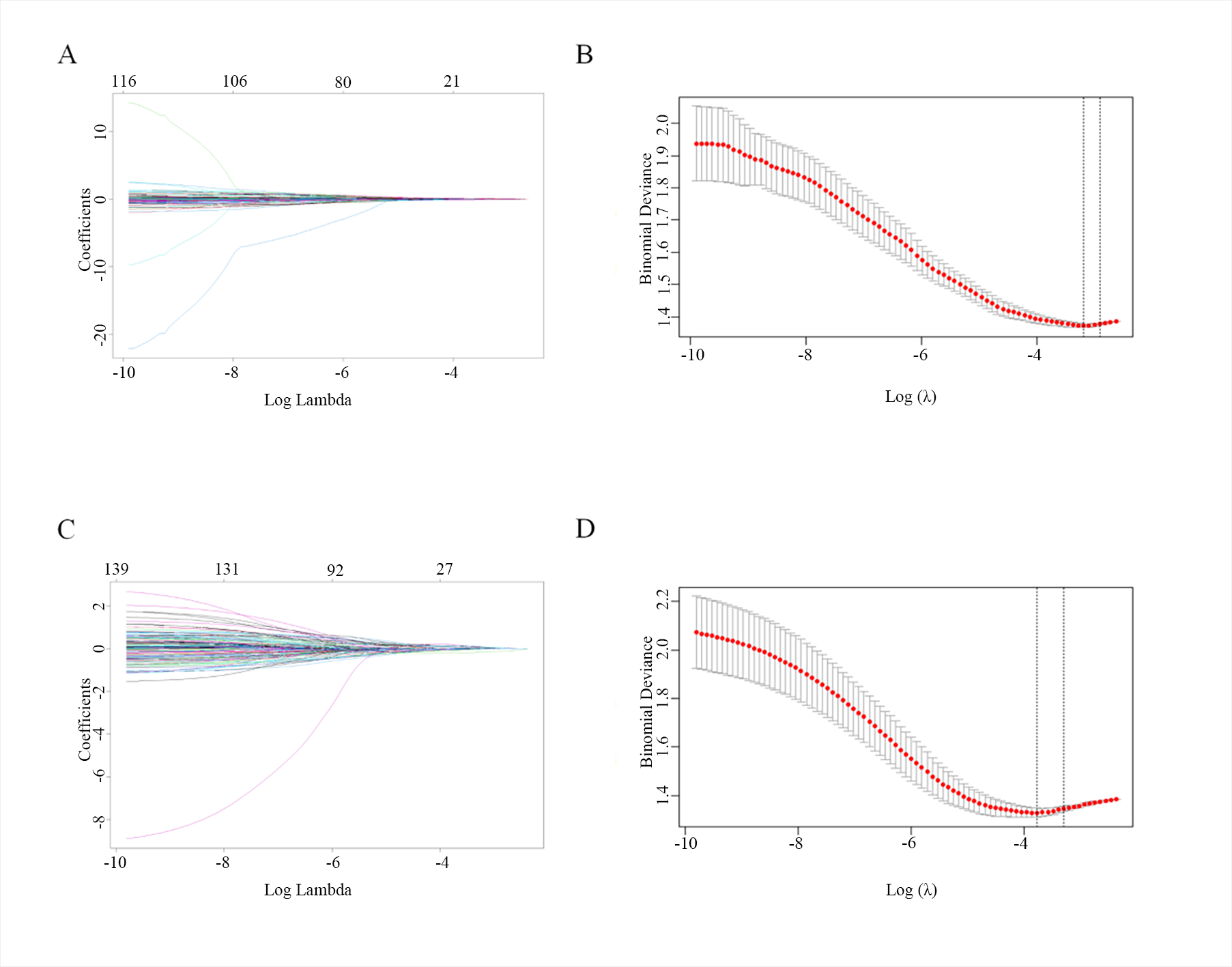


Figure S2 Feature selection using LASSO algorithm. A and B show the cross-validation plot for the penalty term and the LASSO path plot of the HR features. C and D show the cross-validation plot for the penalty term and the LASSO path plot of the SR features. LASSO, least absolute shrinkage and selection operator; HR, high resolution; SR, super resolution.

### Table S2. Selected features by LASSO regression.

| Features | Filter |
| --- | --- |
| HR radiomics features |  |
| First order |  |
| Minimum | LBP-3D-k |
| 10^th^ Percentile | LBP-3D-m1 |
| Texture |  |
| GLSZM_SizeZoneNonUniformity | Wavelet-LLH |
| NGTDM_Complexity | Wavelet-HHL |
| NGTDM_Strength | Wavelet-LLL |
| SR radiomics features |  |
| First order |  |
| Mean | Wavelet-LHL |
| Mean | Wavelet-HLL |
| Minimum | LBP-3D-k |
| Skewness | Wavelet-HLL |
| Texture |  |
| GLCM_ClusterShade | Wavelet-HHL |
| GLCM_Correlation | Lbp-3D-k |
| GLSZM_LargeAreaHighGrayLevelEmphasis | Square |
| GLSZM_LargeAreaLowGrayLevelEmphasis | Square |
| GLSZM_SmallAreaEmphasis | Wavelet-LHH |
| GLSZM_LargeAreaHighGrayLevelEmphasis | Wavelet-LHL |
| GLSZM_ZoneEntropy | Wavelet-HLH |
| GLSZM_LargeAreaHighGrayLevelEmphasis | Wavelet-HHL |
| GLSZM_SmallAreaEmphasis | Wavelet-HHH |
| GLSZM_SmallAreaLowGrayLevelEmphasis | Wavelet-HHH |
| GLSZM_ZoneEntropy | Wavelet-HHH |
| NGTDM_Complexity | Wavelet-HHL |
| NGTDM_Strength | Exponential |
| NGTDM_Strength | Gradient |
| NGTDM_Complexity | Wavelet-LLH |
| NGTDM_Busyness | Wavelet-HLL |

Note.—LBP, Local Binary Patterns; GLSZM, Gray Level Size Zone Matrix; NGTDM, Neighbouring Gray Tone Difference Matrix; GLCM, Gray Level Co-occurrence Matrix.

## 4.2 Classifier selection

Nine classifiers were used to construct models: extreme gradient boosting (XGB), support vector classification (SVC), random forest (RF), multi-layer perceptron (MLP), logistic regression (LR), K-nearest neighbors (KNN), extra trees (ET), decision tree (DT) and adaptive boosting (AdaBoost). Machine learning algorithms are based on scikit-learn (version 1.7, https://scikit-learn.org). Hyperparameter optimization was conducted through stratified five-fold cross-validation combined with grid search over predefined parameter spaces, where configurations maximizing the mean area under the receiver operating characteristic curve (AUC) across validation folds were selected as optimal. The optimal hyperparameters are shown in Table S3.

### Table S3. Optimal Hyperparameter Configurations.

| Classifier | Key Hyperparameters | Value/Range |
| --- | --- | --- |
| Extreme Gradient Boosting | n estimators, max depth, learning rate, colsample bytree, subsample | 500, 5, 1, 0.5, 0.5 |
| Support Vector Classification | C, gamma, kernel | 10, 0.01, rbf |
| Random Forest | n estimators | 8 |
| Multilayer Perceptron | hidden layer sizes, activation, solver, alpha | (50, 50), tanh, sgd, 0.1 |
| Logistic Regression | C, penalty, solver | 0.001, l2, liblinear |
| K-Nearest Neighbors | metric, n neighbors, weights | manhattan, 3, distance |
| Extremely Randomized Trees | max features, n estimators, criterion, max depth, min samples split | log2, 200, gini, 8, 2 |
| Decision Tree | criterion, max depth, min samples split | entropy, 8, 2 |
| Adaptive Boosting | learning rate, n estimators | 0.1, 100 |

# References

1. Ledig C, Theis L, Huszar F, et al. Photo-Realistic Single Image Super-Resolution Using a Generative Adversarial Network. 2017 IEEE Conf Comput Vis Pattern Recognit CVPR. Honolulu, HI: IEEE;2017. p. 105-11. doi: 10.1109/CVPR.2017.19.
2. Wang Z, Bovik AC, Sheikh HR, Simoncelli EP. Image Quality Assessment: From Error Visibility to Structural Similarity. IEEE Trans on Image Process. 2004;13(4):600–612. doi: 10.1109/TIP.2003.819861.
3. Tamang LD, Kim B-W. Super-Resolution Ultrasound Imaging Scheme Based on a Symmetric Series Convolutional Neural Network. Sensors. 2022;22(8):3076. doi: 10.3390/s22083076.
